# *P*-values as percentiles. Commentary on: “Null hypothesis significance tests. A mix–up of two different theories: the basis for widespread confusion and numerous misinterpretations”

**DOI:** 10.3389/fpsyg.2015.00341

**Published:** 2015-04-01

**Authors:** Jose D. Perezgonzalez

**Affiliations:** Business School, Massey UniversityPalmerston North, New Zealand

**Keywords:** *p*-value, probability, percentile, statistical misinterpretations

Schneider's (2015) article is contemporary work addressing the shortcomings of null hypothesis significance testing (NHST). It summarizes previous work on the topic and provides original examples illustrating NHST-induced confusions in scientometrics. Among the confusions cited are those associated with the interpretation of *p*-values, old misinterpretations already investigated by Oakes (1986), Falk and Greenbaum (1995); Haller and Krauss (2000), and Perezgonzalez (2014a), and discussed in, for example, Carver (1978); Nickerson (2000), Hubbard and Bayarri (2003); Kline (2004), and Goodman (2008). That they are still relevant in recent times testifies to the fact that the lessons of the past have not been learnt.

As the title anticipates, there is a twist to this saga, a pedagogical one: *p*-values are typically taught and presented as probabilities, and this may be the cause behind the confusions. A change in the heuristic we use for teaching and interpreting the meaning of *p*-values may be all we need to start working the path toward clarification and understanding.

In this article I will illustrate the differences in interpretation that a percentile heuristic and a probability one make. As guiding example, I will use a one-tailed *p*-value in a normal distribution—*z* = −1.75, *p* = 0.04; Figure 1). The default testing approach will be Fisher's tests of significance, but Neyman–Pearson's tests of acceptance approach will be assumed when discussing Type I errors and alternative hypotheses (for more information about those approaches see Perezgonzalez, 2014b, 2015). The scenario is the scoring of a sample of suspected schizophrenics on a validated psychological normality scale. The hypothesis tested (Fisher's H_0_, Neyman–Pearson's H_M_) is that the mean score of the sample on the normality scale does not differ from that of the normal population (no H_0_ = the sample does not score as normal; H_A_ = the sample scores as schizophrenic, assuming previous knowledge that schizophrenics score low on the scale, by a given effect size). Neither a level of significance nor a rejection region is needed for the discussion.

**Figure 1:**
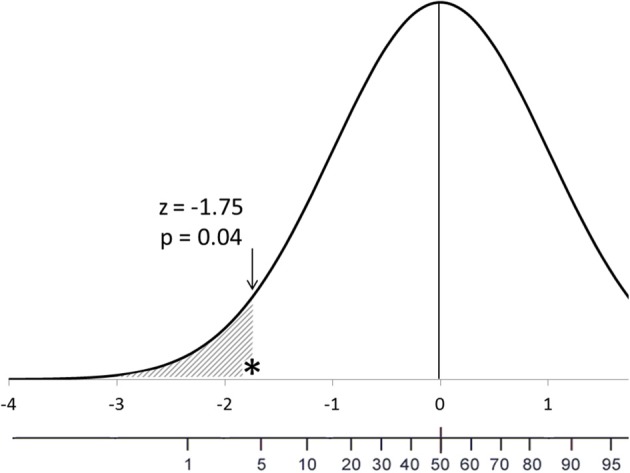
**Location of an observed *z*-score and its corresponding *p*-value in the frequency distribution of the hypothesis under test**. The accompanying scales are for the theoretical *z*-scores and percentiles, respectively.

## *P*-values: probabilities or percentiles?

Let's start by establishing that *p*-values can be interpreted as probabilities. That is, when hypothetical population distributions are generated from sampling data, those frequency distributions follow the frequentist approach and the associated *p*-values show the appropriate probabilities. This is so because these *p*-values are theoretical—they represent the probability of, for example, a hypothetical human being alive today.

The *p*-value we obtain from our research data, however, is not a theoretical, probabilistic, value, but an observed one: its probability of occurrence is “1,” precisely because it has occurred—it represents, for example, the realization that I am alive, not the probability of me being so. Therefore, the observed *p*-value does not represent a probability but a location in the distribution of reference. Among measures of location, percentiles (i.e., percentile ranks) are good heuristics to represent what observed *p*-values really are.

## *P*-values' correct and incorrect misinterpretations

As Figure 1 shows, a percentile describes a fact: the sample scored in the 4th percentile. As a probability, however, the *p*-value is often misinterpreted as, the observed result has a 4% likelihood of having occurred by chance—the *odds-against-chance fantasy* (Carver, 1978)—which also elicits a further misinterpretation as, the observed result has a 96% likelihood of being a real effect (Kline, 2004).

The percentile heuristic also conveys the correct interpretation of the *p*-value as a cumulative percentage in the tail of the distribution: 4% of normal people will score this low or lower. As a probability, the *p*-value is often misinterpreted as, the sample has only a 4% likelihood of being normal—the *inverse probability error* (Cohen, 1994).

Consequently, because the percentile only provides information about location in the distribution of the normal scores hypothesis, it is impossible to know the probability of making a mistake if this hypothesis is rejected. As a probability, the *p*-value is often misinterpreted as, there is only a 4% likelihood of making a mistake when rejecting the tested hypothesis. This is further confused as, the probability of making a Type I error in the long run (alpha, α) is 4%; which then leads to the belief that α can be adjusted a posteriori—roving α (Goodman, 1993)—as a lower than anticipated Type I error (Kline, 2004; Perezgonzalez, 2015).

Furthermore, the percentile is circumscribed to its hypothesis of reference—normal scores on the normality test—and makes no concession for non-tested hypotheses. As a probability, the *p*-value is often misinterpreted as, there is a 96% likelihood that the sample scored as not normal—Fisher's negation of H_0_, the *valid research hypothesis fantasy* (Carver, 1978)—or scored as schizophrenic—Neyman–Pearson's H_A_, the *validity fallacy* (Mulaik et al., 1997).

Finally, the percentile heuristic helps ameliorate misinterpretations regarding future replicability, if only because we normally have enough experience with percentiles in other spheres of life as to realize that the big fish in this pond is neither necessarily big all the time nor equally big in all ponds. As a probability, the *p*-value is often misinterpreted as, there is a 96% likelihood that similar samples will score this low in future studies—the *replicability or reliability fallacy* (Carver, 1978).

## Conclusions

The percentile heuristic is a more accurate model both for interpreting observed *p*-values and for preventing probabilistic misunderstandings. The percentile heuristic may also prove to be a better starting point for demystifying related statistical issues—such as the relationship among *p*-value, effect size and sample size—and epistemological issues—such as statistical significance, and the proving and disproving of hypotheses. All in all, the percentile heuristic matters for better statistical literacy and better research competence, allows for clearer understanding without imposing unnecessary cognitive workload, and has a positive effect in fostering the teaching and practice of psychological science.

### Conflict of interest statement

The author declares that the research was conducted in the absence of any commercial or financial relationships that could be construed as a potential conflict of interest.
